# Wall Shear Stress Distribution in Intracranial Atherosclerotic Disease and Associations With Vessel and Plaque Morphology

**DOI:** 10.1002/cns.70690

**Published:** 2026-01-12

**Authors:** Linfang Lan, Shuang Li, Haipeng Liu, Vincent H.L. Ip, Jill Abrigo, Xuan Tian, Yuying Liu, Yu Liu, Ziqi Li, Tingjun Liang, Florence S. Y. Fan, Sze Ho Ma, Karen Ma, Bonaventure Y.M. Ip, Yannie O. Y. Soo, Howan Leung, Vincent C. T. Mok, Hui Fang, Bo Song, Yuming Xu, Yuhua Fan, Thomas W. Leung, Xinyi Leng

**Affiliations:** ^1^ Department of Neurology, The First Affiliated Hospital Sun Yat‐Sen University, Guangdong Provincial Key Laboratory of Diagnosis and Treatment of Major Neurological Diseases, National Key Clinical Department and Key Discipline of Neurology Guangzhou China; ^2^ Department of Neurology Ren Ji Hospital, Shanghai Jiao Tong University School of Medicine Shanghai China; ^3^ Department of Medicine and Therapeutics The Chinese University of Hong Kong, Prince of Wales Hospital Hong Kong China; ^4^ Research Centre of Intelligent Healthcare Faculty of Health and Life Science, Coventry University Coventry UK; ^5^ National Medical Research Association Leicester UK; ^6^ Cardiovascular Analytics Group PowerHealth Research Institute Hong Kong SAR China; ^7^ Department of Imaging and Interventional Radiology The Chinese University of Hong Kong Hong Kong SAR China; ^8^ Department of Neurology First Affiliated Hospital of Zhengzhou University, NHC Key Laboratory of Prevention and Treatment of Cerebrovascular Disease, Henan Key Laboratory of Cerebrovascular Diseases, Zhengzhou University Zhengzhou China

**Keywords:** computational fluid dynamics, intracranial atherosclerotic disease, wall shear stress

## Abstract

**Background and Aims:**

Wall shear stress (WSS) may govern the initiation and progression of atherosclerosis. We aimed to depict WSS distribution in symptomatic, atherosclerotic M1 middle cerebral artery (MCA‐M1) stenosis, and its associations with adjacent vessel and plaque geometry.

**Methods:**

Patients with symptomatic, atherosclerotic, 50%–99% MCA‐M1 stenosis were analyzed. MCA‐M1 vessel curve orientation and tortuosity, luminal stenosis, plaque length and longitudinal asymmetry were assessed on CT angiography (CTA). Relative WSS (rWSS) was calculated by the absolute WSS divided by mean WSS at the proximal, normal vessel segment, in a CTA‐based computational fluid dynamics model. rWSS < 1.0, 1.0–3.0, and > 3.0 were respectively defined as low, normal, and high WSS; low‐ and high‐WSS areas were measured. The vessel and plaque geometry was associated with the rWSS measures, across a plaque as a whole, and separately in upstream and downstream plaque segments divided at the stenotic throat.

**Results:**

In 176 patients, rWSS increased progressively along the upstream plaque segment but highly varied downstream. rWSS was lower on the inner than on the outer wall of the MCA‐M1 vessel curve. Patients with ventrally (than dorsally), inferiorly (than superiorly) oriented MCA‐M1 vessel curves and higher tortuosity of the affected vessel segment exhibited lower rWSS and larger low‐WSS areas at the downstream plaque segment. More severe luminal stenosis and upstream dominance in the plaque were associated with higher rWSS and larger high‐WSS areas in the upstream and downstream plaque segments.

**Conclusions:**

Wall shear stress (WSS) distribution across symptomatic MCA‐M1 stenosis was variable and strongly associated with adjacent vessel and plaque geometry, independent of systemic factors.

## Graphical abstract

In symptomatic, atherosclerotic middle cerebral artery stenosis: Relative WSS (rWSS) increased progressively along upstream plaque segment but highly varied downstream. Relative WSS (rWSS) was lower on the inner than outer wall of the MCA‐M1 vessel curve. Ventrally and inferiorly oriented middle cerebral artery trunk and higher tortuosity of the vessel segment exhibited lower wall shear stress. Severely stenosed and upstream‐dominant plaque were associated with higher wall shear stress.

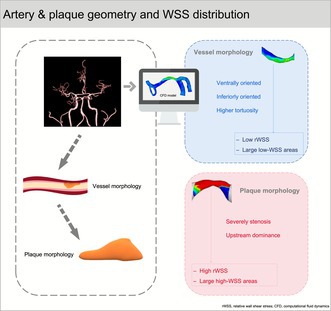

## 1 Introduction

Intracranial atherosclerotic disease (ICAD) is a common cause of ischemic stroke and transient ischemic attack (TIA), particularly in Asia [1]. As a part of systemic atherogenesis, conventional cardiovascular risk factors (e.g., older age, hypertension) [2] and genetic factors [3] all play important roles in ICAD development and progression. However, ICAD is usually anatomically focalized, which means the geometry of adjacent arterial segments and the subsequent hemodynamic environment could also play important roles in the process on top of systemic risk factors.

Wall shear stress (WSS) is a mechanical frictional force generated by blood flow acting on the vessel wall, widely considered a critical hemodynamic force affecting initiation, progression and transformation of atherosclerotic plaques [4, 5]. Previous studies have associated WSS metrics with adjacent arterial geometry in carotid or coronary artery atherosclerosis [6, 7]. However, the flow patterns, WSS distribution and these associations in ICAD (i.e., largely unknown) could be different, as carotid/coronary arteries are mostly straight but intracranial arteries are more tortuous with sharper turnings at bifurcations. Answering these questions in symptomatic ICAD patients could help understand how and why WSS metrics were associated with ICAD lesion progression and stroke recurrence in previous studies [8, 9]. We therefore conducted this study, using a CT angiography (CTA)–based computational fluid dynamics (CFD) model to simulate blood flow and quantify the WSS metrics.

## 2 Patients Methods

### 2.1 Study Design and Subjects

This was a retrospective, cross‐sectional study based on a cohort study, the stroke risk and hemodynamics in intracranial atherosclerotic disease (SOpHIA) study, which recruited acute ischemic stroke and TIA patients with symptomatic ICAD of 50%–99% stenosis confirmed in routine, single‐phase CTA covering from the skull base to the cortex region in a helical mode with no tilting, and a slice thickness of 0.5–0.6 mm. Potentially eligible patients for SOpHIA, with symptomatic M1 middle cerebral artery (MCA‐M1) stenosis and a successfully constructed CTA‐based CFD model, were analyzed in the current study for the associations of the geometric features of MCA‐M1 and the plaque with WSS measures on the symptomatic side. This study was approved by the Joint Chinese University of Hong Kong–New Territories East Cluster Clinical Research Ethics Committee (reference number: 2014.329), with informed consent from all participants. We collected demographics, history of cardiovascular risk factors, blood pressure and NIH stroke scale (NIHSS) at admission, and laboratory test results during hospitalization.

### 2.2 Geometric Features of the Diseased MCA‐M1 and the Plaque in CTA

**FIGURE 1:**
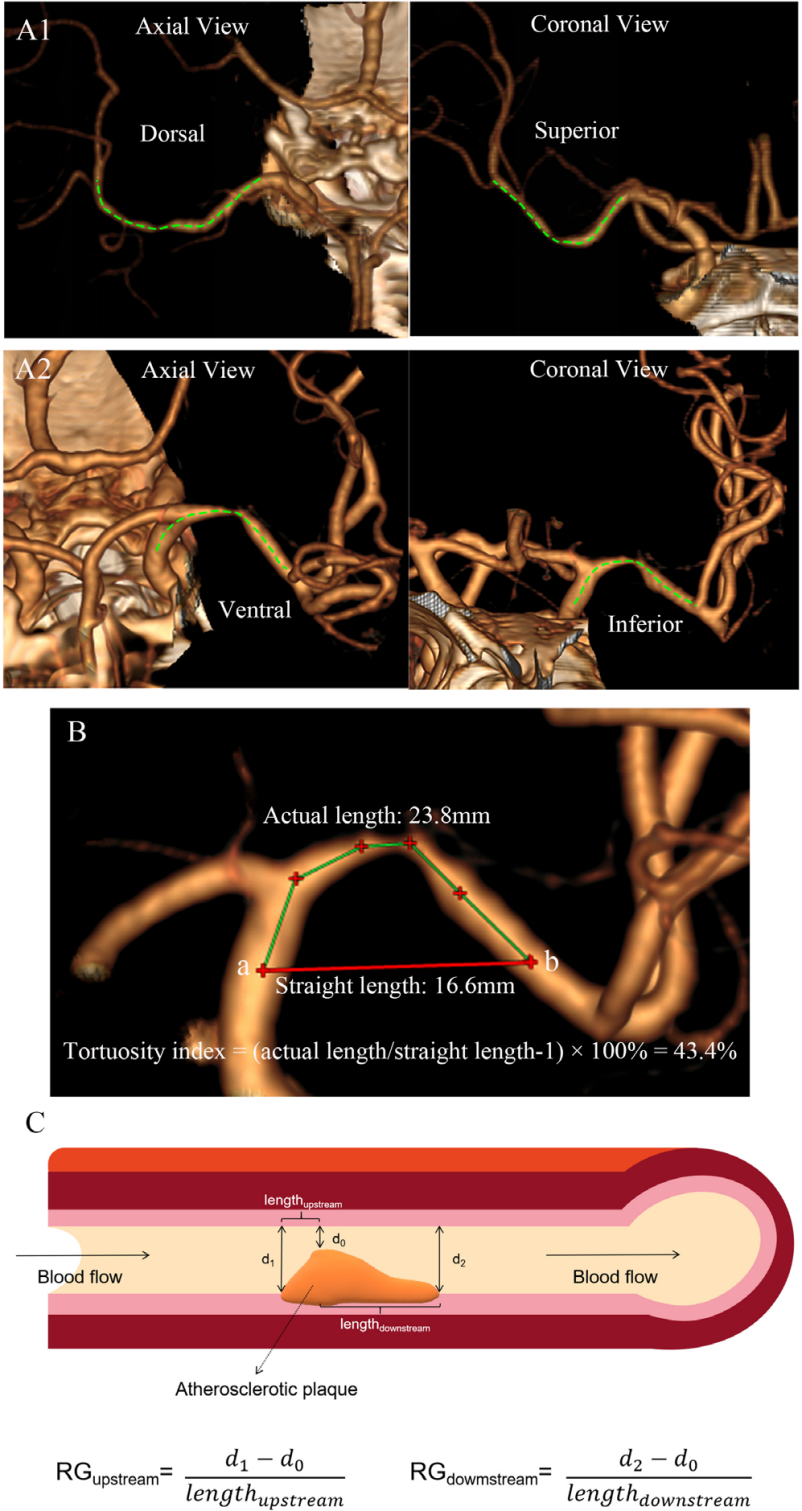
Assessing geometric features of the diseased MCA‐M1 and the plaque. (A) Vessel curve orientation of MCA‐M1 is determined by the direction to which the curve opens, for example, dorsally and superiorly oriented MCA‐M1 in case A1, and ventrally and inferiorly oriented MCA‐M1 in case A2. (Green dotted line: centerline of the arterial trunk). (B) Tortuosity index of the diseased vessel segment = (actual length/straight length—1) × 100%. Green line represents the actual length and red line represents the straight length. (C) RG measurements of the upstream and downstream plaque segments; this is an upstream‐dominant lesion, with RG_upstream_>RG_downstream_. MCA‐M1, M1 segment of middle cerebral artery; RG, radius gradient.

For the diseased MCA‐M1, we defined the vessel curve as ventrally or dorsally oriented in the axial view of CTA, and superiorly or inferiorly oriented in coronal‐view images, according to the direction to which the vessel curve opens Figure 1A [10].

We assessed the tortuosity of the vessel segment containing the symptomatic plaque (i.e., the diseased vessel segment): tortuosity index = (actual length/straight length of the diseased vessel segment—1) × 100 [11]. Actual length of the diseased vessel segment was the length between a starting and an ending point along the vessel route, respectively at the first normal diameters proximal to the plaque extending backward by 6 mm (approximating twice of an MCA diameter), and distal to the plaque extending forward by 6 mm. The straight length was the straight‐line distance between these two points (Figure 1B). The measurement was conducted in the CTA plane showing the maximal tortuosity of the diseased vessel segment.

The degree of luminal stenosis in the diseased MCA‐M1 was measured using the Warfarin–Aspirin Symptomatic Intracranial Disease (WASID) method [12] dichotomized as severe (70%–99%) or moderate (50%–69%) stenosis. Plaque length was defined as the actual distance between the first normal diameters proximal and distal to the lesion.

In addition to being assessed as a whole, a symptomatic ICAD lesion was divided into the upstream and downstream segments by the stenotic throat (i.e., the most severely narrowed cross section). Radius gradients (RG) of upstream and downstream plaque segments were used to assess lesion asymmetry in the longitudinal axis [13, 14] (Figure 1C). Length of upstream plaque segment (length_upstream_), first normal diameter proximal to the lesion (*d*_1_) and diameter at the stenotic throat (*d*_0_) were recorded; RG_upstream_ was calculated as (*d*_1_‐*d*_0_)/length_upstream_; similarly for RG_downstream_. A higher RG indicates a steeper plaque shoulder. With RG_upstream_ > RG_downstream_, the lesion was defined as upstream dominant; otherwise as downstream dominant.

Two investigators (L.L and S.L) with 8 and 5 years of experience assessed the geometric features of the diseased MCA‐M1 and the plaque in three‐dimensional, reconstructed, single‐phase CTA images, using RadiAnt DICOM Viewer (Medixant, Poland), with substantial inter‐rater reliability in 10 cases for these parameters as described in Table S1.

### 2.3 WSS Metrics in CTA‐Based CFD Models

CT angiography (CTA)–based CFD models were constructed to simulate blood flow and quantify WSS in the vicinity of an ICAD lesion, using the ANSYS software package (ANSYS Inc., Canonsburg, PA, USA). The methodology was described previously [8, 9] and briefly in Supplemental Methods.

We measured absolute WSS and rWSS metrics in the CFD models. The rWSS measures were used in the primary analyses, as they could offset intersubject variations in absolute WSS values that could be affected by systemic factors rather than adjacent vessel and plaque geometry that were investigated in this study. The absolute WSS measures were used for sensitivity analyses.

Relative WSS (rWSS) at any location was defined as the ratio of absolute WSS at that location and mean WSS measured at the cross section of a non‐tortuous segment of distal ICA. rWSS < 1.0, 1.0–3.0 and > 3.0 were respectively defined as low, normal and high WSS [5, 9]. rWSS metrics were measured throughout the plaque, and separately in upstream and downstream plaque segments: maximum & minimum rWSS, relative areas of high‐WSS & low‐WSS regions, and the mean rWSS of the high‐WSS & low‐WSS regions. The relative area of high‐ or low‐WSS region was calculated as the percentage of area with high or low WSS, in the whole area of the plaque, and separately in upstream and downstream plaque segments. We assessed the predominance of high‐ or low‐WSS regions in the ventral or dorsal wall of MCA‐M1 based on the CFD model in axial view, and in the superior or inferior vessel wall based on the coronal view. For instance, in axial view, we considered low WSS predominant in the ventral wall, with > 50% of low‐WSS regions located in the ventral wall. For the absolute WSS measures, we recorded the maximum, minimum and mean WSS throughout the plaque, and separately in upstream and downstream plaque segments.

### 2.4 Statistical Analyses

Variables were presented with medians (interquartile range [IQR]) and numbers (percentage). rWSS measures at upstream and downstream plaque segments were compared using Wilcoxon signed‐rank tests. Continuous variables were compared between two groups using Wilcoxon rank sum tests. Correlations between the geometry parameters and WSS measures were assessed using Spearman's correlation coefficients. Sensitivity analyses on absolute WSS measures were conducted with similar methods while fewer parameters were analyzed. Statistical significance was set at a two‐tailed *p*‐value of < 0.05. All statistical analyses were performed using IBM SPSS software version 20.0 (Chicago, USA).

## 3 Results

### 3.1 Patients' Characteristics

**FIGURE 2:**
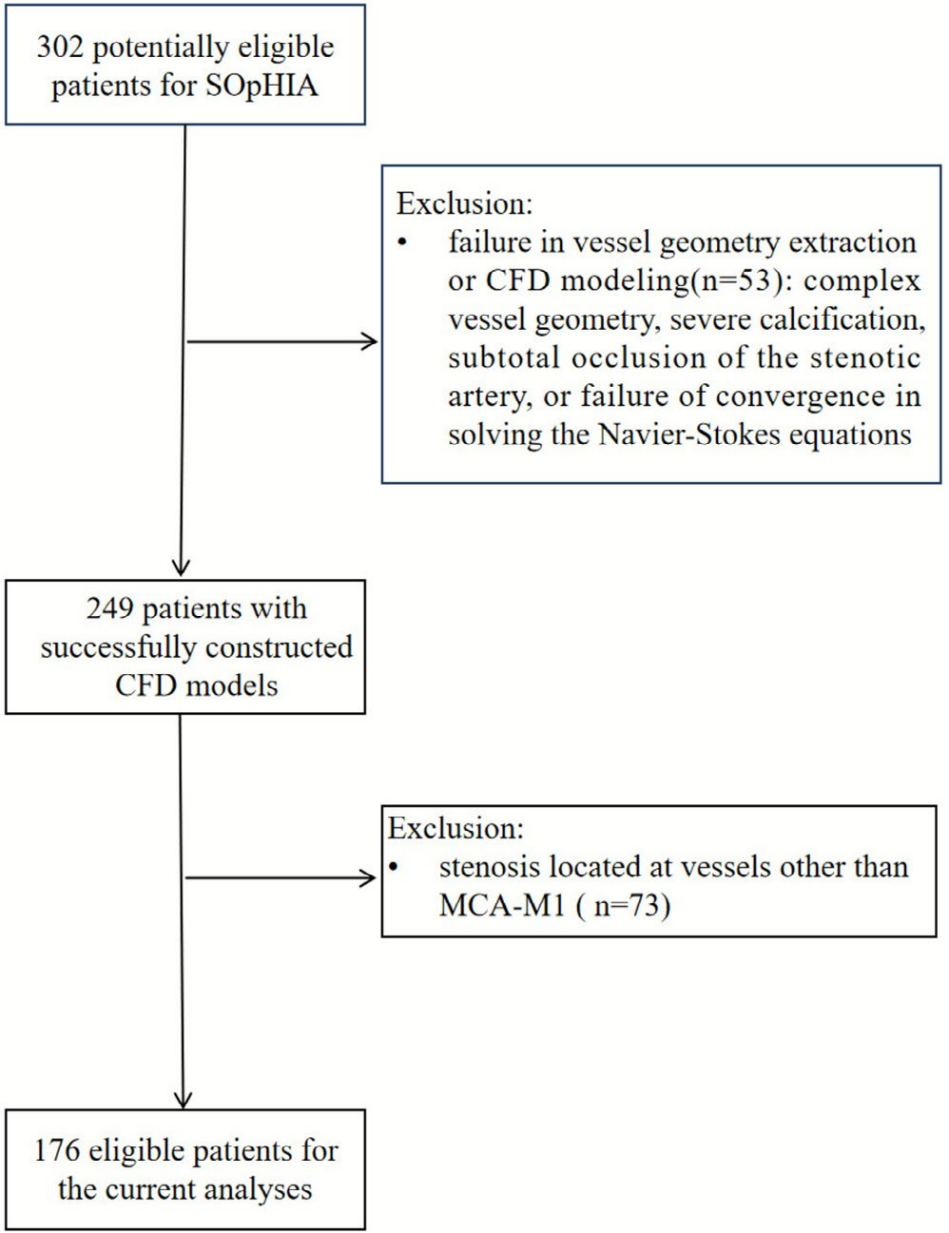
Flowchart of the study. SOpHIA = stroke risk and hemodynamics in intracranial atherosclerotic disease, CFD = computational fluid dynamics, MCA‐M1 = M1 segment of middle cerebral artery.

Among 302 patients who were potentially eligible for the SOpHIA study, 53 were excluded due to complex vessel geometry, severe calcification, subtotal occlusion of the stenotic artery or poor CTA image quality that prevented accurate vessel geometry extraction or failure in CFD modeling; 73 were further excluded due to the ICAD location (Figure 2). The remaining 176 patients were analyzed in the current study, with a median age of 61 (53–70) years, and 123 (70%) males. Demographics and other baseline characteristics are presented in Table 1.

**TABLE 1:** Baseline characteristics.

Characteristics	*n* = 176
Age, years	61 (53–70)
Male	123 (69.9)
SBP at admission, mmHg	152 (134–167)
DBP at admission, mmHg	82 (73–92)
NIHSS at admission	3 (1–4)
History of cardiovascular risk factors	
Hypertension	113 (64.2)
Diabetes	57 (32.4)
Dyslipidemia	102 (58.0)
Smoking	80 (45.5)
Lab test results at baseline	
Fasting glucose, mmol/L	5.5 (4.9–7.0)
HbA1c, %	6.0 (5.6–7.1)
Triglycerides, mmol/L	1.4 (1.1–1.9)
HDL‐C, mmol/L	1.1 (1.0–1.4)
LDL‐C, mmol/L	3.0 (2.3–3.9)
Geometric features the diseased MCA‐M1 and the plaque	
Ventrally/dorsally oriented MCA‐M1 curve	104 (59.1)/72 (40.9)
Inferiorly/superiorly oriented MCA‐M1 curve	111 (63.1)/65 (36.9)
Tortuosity index of the diseased vessel segment, %	13.5 (8.6–22.1)
Percentage of MCA‐M1 luminal stenosis, %	70 (58–78)
Severe stenosis, %	91 (51.7)
Plaque length, mm	5.9 (4.7–7.9)
Upstream/downstream‐dominant plaque	93 (52.8)/83 (47.2)

Abbreviations: DBP, diastolic blood pressure; HbA1c, hemoglobin A1c; HDL‐C, high‐density lipoprotein cholesterol; LDL‐C, low‐density lipoprotein cholesterol; MCA‐M1, M1 segment of middle cerebral artery. NIHSS, NIH stroke scale; SBP, systolic blood pressure.

### 3.2 Geometric Features of the Diseased MCA‐M1 and the Plaque

The curve of the diseased MCA‐M1 was more commonly ventrally (59.1%) than dorsally oriented (40.9%), and more commonly inferiorly (63.1%) than superiorly oriented (36.9%). The median tortuosity index of the diseased vessel segment was 13.5% (8.6%–22.1%). The median luminal stenosis in the MCA‐M1 lesions was 70.0% (58.0%–78.0%), with 51.7% (91 of 176) severe stenoses. The median plaque length was 5.9 (4.7–7.9) mm. Around half of the plaques were upstream‐dominant (52.8%) and half downstream‐dominant (47.2%; Table 1).

### 3.3 Relative WSS (rWSS) Distribution in MCA‐M1 Plaques

**FIGURE 3:**
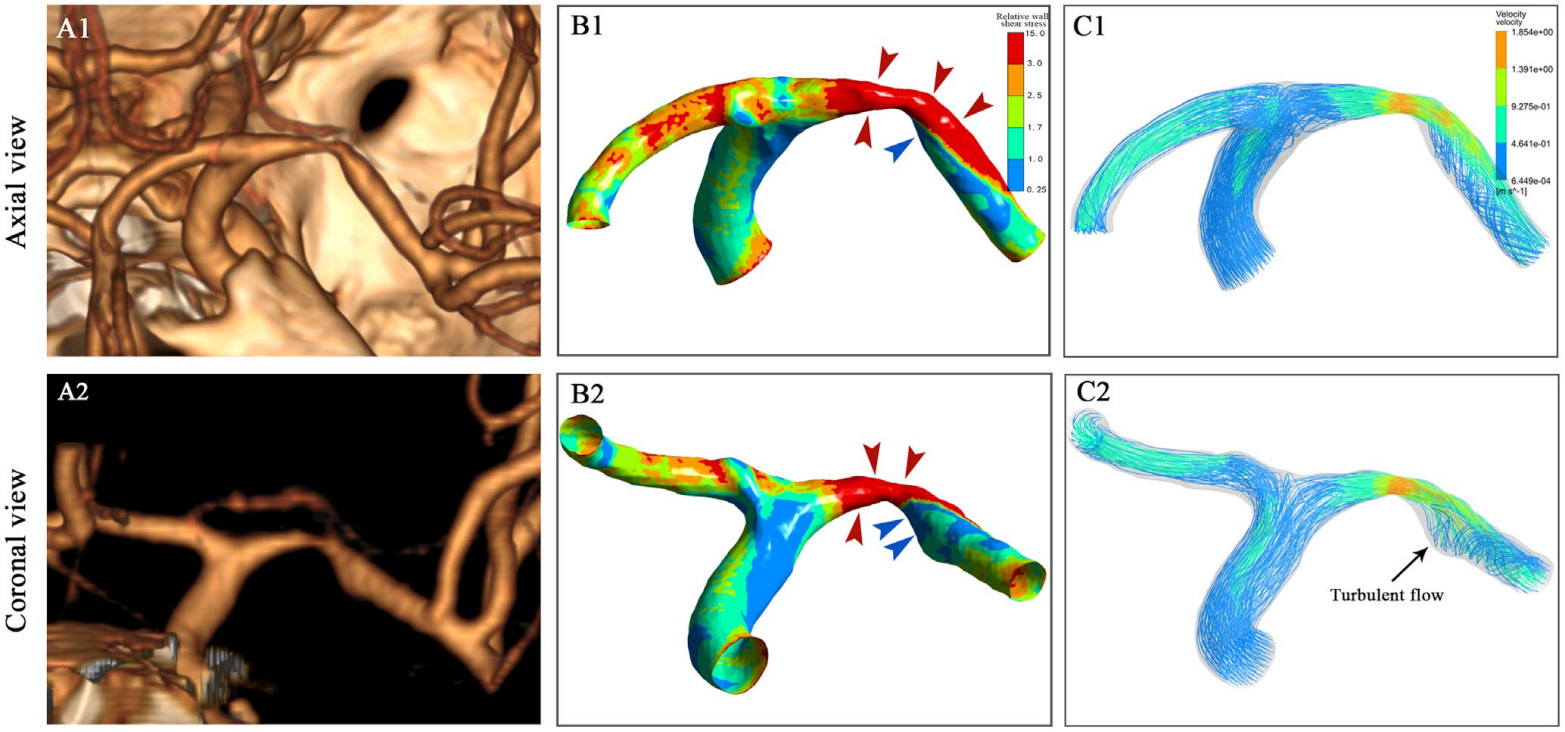
CTA and CFD models in a patient with symptomatic MCA‐M1 stenosis. (A1/A2) Three‐dimensional reconstructed CTA images in the axial and coronal views. (B1/B2) WSS distribution across a stenotic MCA‐M1 lesion illustrated with axial and coronal views. High WSS (rWSS > 3; regions in red color with red arrows) was noticed across the upstream plaque segment, the stenotic throat, and at the outer wall of the vessel curvature at downstream plaque segment; low WSS (rWSS < 1; regions in blue color with blue arrows) was mostly noticed at the inner wall of the vessel curvature at downstream plaque segment. (C1/C2) Significantly increased flow velocity across the stenotic throat, with turbulent and recirculating flow downstream to the lesion. CFD, computational fluid dynamics; CTA, CT angiography; MCA‐M1, M1 segment of middle cerebral artery; rWSS, relative wall shear stress; WSS, wall shear stress.

From the proximal end, rWSS increased progressively along the upstream plaque segment, culminating at the stenotic throat. Yet, rWSS distribution in the downstream plaque segment was more complex, with both high‐ and low‐WSS regions (Figure 3). Compared with that in the upstream plaque segment, the relative area of the low‐WSS region was larger (*p* < 0.001), and the minimum rWSS (*p* < 0.001) and mean rWSS of the low‐WSS region (*p* < 0.001) were lower in the downstream plaque segment. In addition, the mean rWSS of the high‐WSS region was higher (*p* < 0.001) in the downstream plaque segment (Table 2).

**TABLE 2:** Relative wall shear stress (rWSS) measures across symptomatic MCA‐M1 plaques and in the upstream and downstream plaque segments (*n* = 176).

rWSS measures	Throughout the plaque	Upstream plaque segment	Downstream plaque segment	*p*
Maximum rWSS	12.91 (7.01–20.74)	11.73 (6.63–18.69)	12.11 (6.77–20.20)	0.310
Relative area of high‐WSS region	0.54 (0.35–0.68)	0.51 (0.30–0.75)	0.53 (0.34–0.66)	0.292
Mean rWSS of high‐WSS region	6.13 (4.39–9.00)	5.86 (4.29–8.33)	6.42 (4.33–9.59)	< 0.001
Minimum rWSS	0.19 (0.09–0.33)	0.92 (0.55–1.33)	0.19 (0.09–0.34)	< 0.001
Relative area of low‐WSS region	0.09 (0.04–0.14)	0 (0.00–0.02)	0.15 (0.07–0.23)	< 0.001
Mean rWSS of low‐WSS region	0.52 (0.38–0.63)	0.78 (0.65–0.87)	0.48 (0.37–0.63)	< 0.001

*Note:* *p* value was compared between the upstream and downstream plaque segments.

Abbreviations: MCA‐M1, M1 segment of middle cerebral artery; rWSS, relative wall shear stress; WSS, wall shear stress.

In the 104 patients with ventrally oriented MCA‐M1 curve, high WSS was predominant in the dorsal (outer) wall and low WSS was predominant in the ventral (inner) wall of MCA‐M1 in a majority of patients (*n* = 88; 84.6%). Similarly, high WSS was predominant in the outer wall and low WSS was predominant in the inner wall of MCA‐M1 in 53 (73.6%) of the 72 patients with dorsally oriented MCA‐M1 curve, 92 (82.9%) of the 111 patients with inferiorly oriented MCA‐M1 curve, 49 (75.4%) of the 65 patients with superiorly oriented MCA‐M1 curve.

### 3.4 Associations of Vessel and Plaque Geometry With the rWSS Measures

Table 3 shows the associations between the geometric features of MCA‐M1 and the rWSS measures throughout the plaque, as well as separately in the upstream and downstream plaque segments. rWSS throughout the plaque was on average lower in those with a ventrally than dorsally oriented MCA‐M1 curve: lower minimum rWSS (*p* = 0.005) and lower mean rWSS of the low‐WSS region (*p* = 0.006), and larger low‐WSS area (*p* = 0.017). Similar trends were found in those with an inferiorly versus superiorly oriented MCA‐M1 curve, though some did not reach statistical significance. In addition, a higher tortuosity index was associated with lower rWSS: minimum rWSS (*p* = 0.002) and mean rWSS of the low‐WSS region (*p* = 0.001), and larger low‐WSS area (*p* < 0.001). These relationships between MCA‐M1 geometric features and WSS measures were also observed when analyzing the downstream plaque segment separately, but not in the upstream plaque segment. Overall, the vessel curvature orientation and tortuosity of a diseased MCA‐M1 were significantly associated with low‐WSS measures in an MCA‐M1 plaque, predominantly in the downstream plaque segment.

**TABLE 3:** Associations between the geometric features of MCA‐M1 and the rWSS measures.

rWSS measures	Vessel curve orientation of the diseased MCA‐M1 (*n* = 176)						Tortuosity index of the diseased vessel segment (*n* = 176)	
	Ventral (*n* = 104)	Dorsal (*n* = 72)	*p*	Inferior (*n* = 111)	Superior (*n* = 65)	*p*	Spearman R	*p*
Throughout the plaque								
Maximum rWSS	12.79 (6.77–18.87)	13.31 (7.26–23.10)	0.413	12.01 (6.70–19.75)	13.10 (7.54–25.33)	0.214	0.012	0.880
Relative area of high‐WSS region	0.54 (0.35–0.69)	0.55 (0.36–0.68)	0.858	0.50 (0.33–0.68)	0.55 (0.39–0.68)	0.466	0.091	0.233
Mean rWSS of high‐WSS region	5.94 (4.39–8.50)	6.46 (4.41–9.65)	0.463	6.01 (4.29–8.50)	6.46 (4.57–9.55)	0.340	0.008	0.913
Minimum rWSS	0.14 (0.08–0.27)	0.22 (0.14–0.34)	0.005	0.15 (0.08–0.27)	0.22 (0.14–0.40)	0.010	−0.234	0.002
Relative area of low‐WSS region	0.10 (0.04–0.16)	0.07 (0.03–0.11)	0.017	0.09 (0.05–0.15)	0.07 (0.03–0.12)	0.059	0.269	< 0.001
Mean rWSS of low‐WSS region	0.50 (0.35–0.61)	0.57 (0.43–0.68)	0.006	0.51 (0.37–0.61)	0.56 (0.43–0.67)	0.117	−0.249	0.001
Upstream plaque segment								
Maximum rWSS	10.13 (6.48–18.27)	12.14 (7.02–21.38)	0.336	10.17 (6.28–18.36)	12.40 (7.35–21.10)	0.220	0.098	0.196
Relative area of high‐WSS region	0.50 (0.28–0.78)	0.52 (0.31–0.72)	0.777	0.50 (0.26–0.79)	0.54 (0.37–0.72)	0.600	0.160	0.038
Mean rWSS of high‐WSS region	5.34 (4.17–7.45)	6.19 (4.50–9.10)	0.189	5.50 (4.15–8.00)	5.89 (4.55–8.69)	0.325	0.125	0.100
Minimum rWSS	0.95 (0.55–1.39)	0.85 (0.51–1.32)	0.394	0.97 (0.62–1.51)	0.83 (0.47–1.29)	0.085	0.063	0.409
Relative area of low‐WSS region	0 (0–0.01)	0 (0–0.04)	0.430	0 (0–0.01)	0 (0–0.04)	0.170	0.019	0.804
Mean rWSS of low‐WSS region	0.79 (0.65–0.89)	0.76 (0.68–0.84)	0.674	0.79 (0.67–0.90)	0.72 (0.65–0.83)	0.192	0.097	0.402
Downstream plaque segment								
Maximum rWSS	12.50 (6.59–18.32)	11.92 (6.86–22.78)	0.588	12.01 (6.33–19.24)	12.61 (7.19–22.19)	0.312	−0.052	0.495
Relative area of high‐WSS region	0.53 (0.32–0.64)	0.52 (0.39–0.66)	0.676	0.51 (0.32–0.65)	0.57 (0.40–0.68)	0.169	−0.179	0.018
Mean rWSS of high‐WSS region	6.23 (4.13–9.13)	6.52 (4.32–9.94)	0.564	6.18 (4.13–9.13)	6.65 (4.42–10.18)	0.345	−0.080	0.294
Minimum rWSS	0.14 (0.08–0.27)	0.22 (0.14–0.42)	0.003	0.16 (0.08–0.31)	0.22 (0.14–0.42)	0.018	−0.249	0.001
Relative area of low‐WSS region	0.18 (0.08–0.25)	0.12 (0.06–0.19)	0.009	0.17 (0.08–0.25)	0.13 (0.06–0.22)	0.105	0.357	< 0.001
Mean rWSS of low‐WSS region	0.44 (0.33–0.59)	0.53 (0.41–0.67)	0.005	0.44 (0.35–0.61)	0.51 (0.40–0.65)	0.084	−0.194	0.012

Abbreviations: MCA‐M1, M1 segment of middle cerebral artery; rWSS, relative wall shear stress.

Table 4 shows the relationships between morphological features of the MCA‐M1 plaques and the rWSS measures. More severe luminal stenosis was associated with overall higher WSS throughout the plaque, with higher rWSS values, larger high‐WSS area, and smaller low‐WSS area. The plaque length was not associated with the WSS measures, except for the mean rWSS of the low‐WSS region (*p* = 0.042). Upstream‐dominant plaques on average had a higher maximum rWSS, larger high‐WSS area and higher mean rWSS of the high‐WSS region, than downstream‐dominant plaques; but the low‐WSS measures were not significantly different between the two groups. Similar relationships were also observed between the plaque morphology features and the rWSS measures separately in the upstream and downstream plaque segments, except that there was no significant association between the degree of luminal stenosis and low‐WSS measures in the upstream plaque segment. Overall, the degree of luminal stenosis and lesion asymmetry in the longitudinal axis were significantly associated with high‐WSS measures across an MCA‐M1 plaque, as well as separately in the upstream and downstream plaque segments.

**TABLE 4:** Associations between morphological features of the MCA‐M1 plaques and the rWSS measures.

rWSS measures	Percentage of luminal stenosis (*n* = 176)		Plaque length (*n* = 176)		Lesion asymmetry in the longitudinal axis (*n* = 176)		
	Spearman R	*p*	Spearman R	*p*	Upstream‐dominant (*n* = 93)	Downstream‐dominant (*n* = 83)	*p*
Throughout the stenotic lesion							
Maximum rWSS	0.497	< 0.001	0.044	0.563	15.32 (8.67–23.79)	8.44 (5.98–17.93)	0.001
Relative area of high‐WSS region	0.258	0.001	0.020	0.790	0.58 (0.41–0.70)	0.46 (0.28–0.64)	0.004
Mean rWSS of the high‐WSS region	0.478	< 0.001	0.026	0.731	7.22 (4.99–9.72)	5.14 (4.04–8.10)	0.002
Minimum rWSS	0.330	< 0.001	−0.088	0.247	0.21 (0.10–0.34)	0.16 (0.09–0.28)	0.320
Relative area of low‐WSS region	−0.207	0.006	−0.029	0.702	0.09 (0.04–0.13)	0.09 (0.04–0.16)	0.495
Mean rWSS of the low‐WSS region	0.306	< 0.001	−0.156	0.042	0.56 (0.41–0.64)	0.47 (0.36–0.63)	0.088
Upstream plaque segment							
Maximum rWSS	0.451	< 0.001	0.026	0.732	13.78 (8.67–21.52)	7.23 (5.26–16.23)	< 0.001
Relative area of high‐WSS region	0.185	0.014	0.032	0.674	0.61 (0.37–0.79)	0.46 (0.24–0.70)	0.009
Mean rWSS of the high‐WSS region	0.399	< 0.001	0.005	0.948	6.26 (4.85–9.40)	4.55 (3.97–7.10)	< 0.001
Minimum rWSS	0.128	0.090	0.091	0.227	0.89 (0.49–1.48)	0.92 (0.57–1.30)	0.980
Relative area of low‐WSS region	−0.080	0.294	−0.147	0.052	0 (0.00–0.03)	0 (0.00–0.02)	0.533
Mean rWSS of the low‐WSS region	0.064	0.579	0.219	0.054	0.72 (0.65–0.83)	0.79 (0.70–0.89)	0.267
Downstream plaque segment							
Maximum rWSS	0.511	< 0.001	0.075	0.323	14.33 (8.37–22.96)	7.87 (5.74–17.14)	0.001
Relative area of high‐WSS region	0.280	< 0.001	0.066	0.387	0.57 (0.43–0.67)	0.50 (0.26–0.64)	0.012
Mean rWSS of the high‐WSS region	0.487	< 0.001	0.086	0.261	7.31 (4.86–10.69)	5.22 (3.99–8.92)	0.009
Minimum rWSS	0.307	< 0.001	−0.100	0.188	0.21 (0.10–0.34)	0.16 (0.09–0.34)	0.262
Relative area of low‐WSS region	−0.170	0.024	0	0.996	0.15 (0.07–0.22)	0.17 (0.07–0.25)	0.172
Mean rWSS of the low‐WSS region	0.306	< 0.001	−0.129	0.095	0.52 (0.38–0.64)	0.44 (0.34–0.61)	0.133

Abbreviations: MCA‐M1, M1 segment of middle cerebral artery; rWSS, relative wall shear stress.

### 3.5 Sensitivity Analyses

Sensitivity analyses with absolute WSS measures showed similar trends with the findings based on rWSS measures, which were presented in Supplemental Results and Tables S2–S4.

## 4 Discussion

We depicted WSS distribution patterns across symptomatic, atherosclerotic MCA‐M1 stenosis using a CTA‐based CFD model, and explored their relationships with focal vessel geometry and plaque morphology. WSS progressively elevated along the upstream plaque segment, with mixed high‐ and low‐WSS regions downstream. On average, WSS was lower on the inner wall than the outer wall of the MCA‐M1 vessel curve. Patients with ventrally, inferiorly oriented and more tortuous MCA‐M1 vessel curves exhibited lower WSS and larger low‐WSS areas at downstream plaque segments. Moreover, more severe luminal stenosis and upstream dominance in the plaque (i.e., steeper slope at upstream than downstream plaque segments) were associated with higher WSS and larger high‐WSS areas in upstream and downstream plaque segments. Findings were similar from the primary analyses with rWSS measures and sensitivity analyses with absolute WSS measures.

The peaking WSS near the stenotic throat, variable WSS downstream, and the lower WSS on the inner than outer wall of a curved MCA in this study, are consistent with previous carotid or coronary artery studies [6, 15]. In arterial stenosis, upstream WSS would increase with increasing flow velocities, while downstream there could be high‐WSS regions with the flow jet passing through the lesion, and low‐WSS regions with disturbed or oscillatory flow on the opposite wall. In a curved vessel, higher flow velocities near the outer wall due to centrifugal forces could explain the higher WSS on the outer wall.

This study revealed differences in WSS distributions at different parts of the intracranial plaque and adjacent arterial segments, which also varied with different vessel and plaque geometry. This may affect the development and progression of intracranial atherosclerosis at different locations and in different individuals, as WSS is a double‐edged sword in atherosclerosis, with low WSS prone to atherogenesis and high WSS associated with increased plaque vulnerability [5]. For instance, we found lower WSS and larger low‐WSS areas in those with ventrally, inferiorly oriented and more tortuous MCA‐M1 vessel curves. Previous studies have revealed more plaques located in the ventral and inferior walls of the MCA trunk [10, 16], and increased risks of plaque formation and progression in more tortuous intracranial and femoral arteries [11, 17]. These all supported the pro‐atherogenic effect of low WSS, probably by inducing endothelial dysfunction and secondary inflammation [5]. We also found higher WSS and larger high‐WSS areas in upstream‐dominant than downstream‐dominant MCA‐M1 plaques, consistent with findings from coronary artery studies [14]. This may partly explain why almost all upstream plaque ruptures occur in upstream‐dominant coronary plaques [14], as high WSS may increase the necrotic core, thin the fibrous cap, induce expansive remodeling of atherosclerotic plaques, and ultimately lead to increased plaque vulnerability [5]. Moreover, previous studies have also associated high WSS with artery‐to‐artery embolism and a higher risk of stroke relapse in symptomatic ICAD, which reinforced the association between higher WSS and higher plaque vulnerability [8, 18].

Findings of this study could deepen our understanding of the hemodynamic behavior of blood flow adjacent to an intracranial plaque, as governed by the vessel and plaque geometry. The findings also have clinical implications. Previous studies have suggested the roles of WSS measures in affecting the stroke mechanisms in symptomatic ICAD in cross‐sectional studies [18], and progression/regression of luminal stenosis and the risk of stroke relapse under modern medical treatment in longitudinal studies [8, 9]. Findings from the current study and future longitudinal studies on the reciprocal interactions between vessel/plaque geometry and focal hemodynamics over time, could help understand how and why WSS metrics were associated with ICAD lesion progression and stroke recurrence. In addition, although angioplasty +/− stenting was not recommended as first‐line treatment for symptomatic, high‐grade ICAD patients in current guidelines, carefully selected patients may benefit from such therapy [18], while subsequent studies on how angioplasty +/− stenting would alter focal vessel geometry and affect the focal/distal hemodynamic environment may provide some evidence for further testing in clinical trials.

Our study had limitations. First, the study was restricted to Chinese patients with symptomatic, 50%–99% MCA‐M1 stenosis. Further studies are needed to verify the effects of vessel and plaque geometry on the hemodynamics in other stages of atherosclerosis or arteries with different architecture, and in other populations. Moreover, systemic factors such as blood pressure, hematocrit, and cerebral autoregulation could affect focal hemodynamics in ICAD. Yet, we used uniform boundary conditions and blood property assumptions in CFD modeling. This in fact ensured that the associations of vessel and plaque geometry with the WSS measures were independent of the systemic factors. The effects of these factors could be explored in future larg‐scale studies. In addition, we were not able to reveal the dynamic changes of the plaque, WSS measures, or their associations with vessel/plaque geometry, in this cross‐sectional study, which could be investigated in future longitudinal studies. Besides, we did not perform multivariate analysis as there were several dependent variables in the current study. Potential interactions may also exist between different plaque and vessel morphology, which further limits the multiple comparison corrections. Finally, as invasive assessment of WSS and other hemodynamic parameters is not routinely performed in sICAS patients in clinical practice, we have not investigated the consistency of CFD‐simulated hemodynamic parameters with in vivo measurements. Future studies could verify the reliability of CFD‐derived hemodynamic parameters with those obtained with other methods, for example, four‐dimensional flow magnetic resonance imaging.

In conclusion, WSS distribution across symptomatic MCA‐M1 stenosis was complex and heterogeneous, which was strongly associated with the geometric features of the adjacent arterial segments and the plaque. These findings could help in understanding the hemodynamic behavior of blood flow adjacent to an intracranial plaque, as governed by the vessel and plaque geometry, independent of systemic factors. Further longitudinal studies are warranted, to facilitate prediction of where and how intracranial plaques would initiate, grow and rupture, and more importantly and clinically relevant, how these would inform stroke risks and treatments.

## Funding

This work was supported by the Young Scientists Fund (No. 81901187), National Natural Science Foundation of China; Early Career Scheme (Reference No. 24103122), Research Grants Council of Hong Kong; Health and Medical Research Fund (Reference No. 10210366), Hong Kong Food and Health Bureau; Li Ka Shing Institute of Health Sciences; Guangzhou science‐brain project (No. 2023A04J2193), Guangzhou Municipal science and technology.

## Ethics Statement

This study was approved by Joint Chinese University of Hong Kong–New Territories East Cluster Clinical Research Ethics Committee (Reference No.: 2014.329).

## Consent

All patients provided informed consent.

## Conflicts of Interest

The authors declare no conflicts of interest.

## Supporting information


**Data S1:** Supporting Information.

## Data Availability

The data that support the findings of this study are available from the corresponding author upon reasonable request.

## References

[1] X. Leng , R. Hurford , X. Feng , et al., “Intracranial Arterial Stenosis in Caucasian Versus Chinese Patients With TIA and Minor Stroke: Two Contemporaneous Cohorts and a Systematic Review,” Journal of Neurology, Neurosurgery, and Psychiatry 92 (2021): 590–597, 10.1136/jnnp-2020-325630. 33785575 PMC8142447

[2] C. A. Holmstedt , T. N. Turan , and M. I. Chimowitz , “Atherosclerotic Intracranial Arterial Stenosis: Risk Factors, Diagnosis, and Treatment,” Lancet Neurology 12 (2013): 1106–1114, 10.1016/s1474-4422(13)70195-9. 24135208 PMC4005874

[3] M. Shi , X. Leng , Y. Li , et al., “Genome Sequencing Reveals the Role of Rare Genomic Variants in Chinese Patients With Symptomatic Intracranial Atherosclerotic Disease,” Stroke and Vascular Neurology 7, no. 3 (2022): 182–189. 34880113 10.1136/svn-2021-001157 PMC9240611

[4] H. Samady , P. Eshtehardi , M. C. McDaniel , et al., “Coronary Artery Wall Shear Stress Is Associated With Progression and Transformation of Atherosclerotic Plaque and Arterial Remodeling in Patients With Coronary Artery Disease,” Circulation 124, no. 7 (2011): 779–788, 10.1161/circulationaha.111.021824. 21788584

[5] J. J. Wentzel , Y. S. Chatzizisis , F. J. Gijsen , G. D. Giannoglou , C. L. Feldman , and P. H. Stone , “Endothelial Shear Stress in the Evolution of Coronary Atherosclerotic Plaque and Vascular Remodelling: Current Understanding and Remaining Questions,” Cardiovascular Research 96, no. 2 (2012): 234–243, 10.1093/cvr/cvs217. 22752349

[6] M. Markl , F. Wegent , T. Zech , et al., “In Vivo Wall Shear Stress Distribution in the Carotid Artery: Effect of Bifurcation Geometry, Internal Carotid Artery Stenosis, and Recanalization Therapy,” Circulation. Cardiovascular Imaging 3 (2010): 647–655, 10.1161/circimaging.110.958504. 20847189

[7] S. A. Katranas , A. P. Antoniadis , A. L. Kelekis , and G. D. Giannoglou , “Insights on Atherosclerosis by Non‐Invasive Assessment of Wall Stress and Arterial Morphology Along the Length of Human Coronary Plaques,” International Journal of Cardiovascular Imaging 31, no. 8 (2015): 1627–1633, 10.1007/s10554-015-0736-5. 26255177

[8] X. Leng , L. Lan , H. L. Ip , et al., “Hemodynamics and Stroke Risk in Intracranial Atherosclerotic Disease,” Annals of Neurology 85 (2019): 752–764, 10.1002/ana.25456. 30840312

[9] L. Lan , H. Liu , V. Ip , et al., “Regional High Wall Shear Stress Associated With Stenosis Regression in Symptomatic Intracranial Atherosclerotic Disease,” Stroke 51 (2020): 3064–3073, 10.1161/strokeaha.120.030615. 32883193

[10] Y. N. Yu , M. L. Li , Y. Y. Xu , et al., “Middle Cerebral Artery Geometric Features Are Associated With Plaque Distribution and Stroke,” Neurology 91 (2018): e1760–e1769, 10.1212/wnl.0000000000006468. 30291186

[11] B. J. Kim , S. M. Kim , D. W. Kang , S. U. Kwon , D. C. Suh , and J. S. Kim , “Vascular Tortuosity May Be Related to Intracranial Artery Atherosclerosis,” International Journal of Stroke 10 (2015): 1081–1086, 10.1111/ijs.12525. 26061533

[12] O. B. Samuels , G. J. Joseph , M. J. Lynn , H. A. Smith , and M. I. Chimowitz , “A Standardized Method for Measuring Intracranial Arterial Stenosis,” AJNR. American Journal of Neuroradiology 21 (2000): 643–646. 10782772 PMC7976653

[13] G. Choi , J. M. Lee , H. J. Kim , et al., “Coronary Artery Axial Plaque Stress and Its Relationship With Lesion Geometry: Application of Computational Fluid Dynamics to Coronary CT Angiography,” JACC: Cardiovascular Imaging 8 (2015): 1156–1166, 10.1016/j.jcmg.2015.04.024. 26363834

[14] J. M. Lee , G. Choi , D. Hwang , et al., “Impact of Longitudinal Lesion Geometry on Location of Plaque Rupture and Clinical Presentations,” JACC: Cardiovascular Imaging 10 (2017): 677–688, 10.1016/j.jcmg.2016.04.012. 27665158

[15] K. K. L. Wong , J. Wu , G. Liu , W. Huang , and D. N. Ghista , “Coronary Arteries Hemodynamics: Effect of Arterial Geometry on Hemodynamic Parameters Causing Atherosclerosis,” Medical & Biological Engineering & Computing 58 (2020): 1831–1843, 10.1007/s11517-020-02185-x. 32519006

[16] W. H. Xu , M. L. Li , S. Gao , et al., “Plaque Distribution of Stenotic Middle Cerebral Artery and Its Clinical Relevance,” Stroke 42 (2011): 2957–2959, 10.1161/strokeaha.111.618132. 21799160

[17] O. Smedby and L. Bergstrand , “Tortuosity and Atherosclerosis in the Femoral Artery: What Is Cause and What Is Effect?,” Annals of Biomedical Engineering 24 (1996): 474–480, 10.1007/bf02648109. 8841722

[18] X. Feng , H. Fang , B. Ip , et al., “Cerebral Hemodynamics Underlying Artery‐To‐Artery Embolism in Symptomatic Intracranial Atherosclerotic Disease,” Translational Stroke Research 15, no. 3 (2023): 572–579, 10.1007/s12975-023-01146-4. 36897543

