# Supplementary material for: Wall Shear Stress Distribution in Intracranial Atherosclerotic Disease and Associations With Vessel and Plaque Morphology
Source: CNS Neurosci Ther. 2026 Jan 12;32(1):e70690. doi: 10.1002/cns.70690 (PMC12794665; doi:10.1002/cns.70690)
Supplement: Supplementary file 1 — Data S1: Supporting Information. [file CNS-32-e70690-s001.docx]

**Table of contents**

[Supplemental methods 2](#_Toc175624689)

[Supplemental results 3](#_Toc175624690)

[Supplemental Table 1 4](#_Toc175624691)

[Supplemental Table 2 5](#_Toc175624692)

[Supplemental Table 3 6](#_Toc175624693)

[Supplemental Table 4 7](#_Toc175624694)

**Supplemental methods on CT angiography (CTA)-based computational fluid dynamics (CFD) modeling**

The detailed methodology of CTA-based CFD modeling was described previously (1, 2). Briefly, the 3D geometry of the arteries of interest was reconstructed from CTA. A mesh was created on the vessel surface and lumen, with a maximal element size of 0.1 for the inlet/outlets, and 0.25 for other parts. A mean blood pressure of 110 mmHg was set at the internal carotid artery ICA inlet; mass flow rates were applied at the outlets, estimated based on flow velocities from a population-based study (3). Blood flow simulation was then performed by solving the Navier-Stokes equations, assuming rigid and non-compliant arterial wall, with a no-slip flow condition, and the blood as an incompressible Newtonian ﬂuid with a constant viscosity of 0.0035 $kg\cdot m^{-1}\cdot s^{-1}$ and a density of 1,060 $kg\cdot m^{-3}$. CFD modeling and hemodynamic parameter measurements were reproducible (1).

**Supplemental references**

1. Leng X, Lan L, Ip HL, et al. Hemodynamics and stroke risk in intracranial atherosclerotic disease. Ann Neurol 2019; 85(5):752-764.

2. Lan L, Liu H, Ip V, et al. Regional high wall shear stress associated with stenosis regression in symptomatic intracranial atherosclerotic disease. Stroke 2020; 51(10):3064-3073.

3. Tegeler CH, Crutchfield K, Katsnelson M, et al. Transcranial Doppler velocities in a large, healthy population. J Neuroimaging 2013; 23(3):466-472.

**Supplemental results**

First, the downstream plaque segments had lower minimum WSS (p<0.001) but comparable maximum WSS, compared with the upstream plaque segments (Supplemental Table 2). Second, there were numerically or statistically significant lower minimum WSS in those with a ventrally than dorsally oriented MCA-M1 curve and in those with an inferiorly versus superiorly oriented MCA-M1 curve, and a higher tortuosity index of the diseased vessel segment was associated with lower minimum WSS; these were observed throughout the plaque and in the downstream plaque segment but not in the upstream plaque segment (Supplemental Table 3). Third, more severe luminal stenosis was associated with higher maximum, minimum and mean WSS values, and upstream-dominant plaques had higher maximum and mean WSS values than downstream-dominant plaques, throughout the plaque and separately in the upstream and downstream plaque segments (Supplemental Table 4).

**Supplemental Table 1.** Interobserver agreement of measurements of geometric features

| **Geometric features** | Interobserver agreement  Kappa (p) or ICC (95% CI) |
| --- | --- |
| Ventral- and dorsal-orientation | 0.737 (0.016) |
| Superior- and inferior-orientation | 0.783 (0.011) |
| Tortuosity index | 0.912 (0.686-0.977) |
| Percentage of MCA-M1 luminal stenosis | 0.946 (0.782-0.987) |
| Plaque length | 0.845 (0.375-0.961) |
| Radius gradient | 0.803 (0.390-0.947) |

MCA-M1, M1 segment of middle cerebral artery; ICC, intraclass correlation coefficient.

**Supplemental Table 2.** Absolute WSS measures across symptomatic MCA-M1 plaques and in the upstream and downstream plaque segments (n=176)

| **Absolute** **WSS measures** | Throughout  the plaque | Upstream  plaque segment | Downstream plaque segment | P value |
| --- | --- | --- | --- | --- |
| Maximum WSS | 41.12 (27.29-67.62) | 38.69 (23.47-59.87） | 39.87 (25.38-61.97) | 0.167 |
| Minimum WSS | 0.58 (0.31-0.96) | 2.97 (1.81-4.74) | 0.61 (0.32-1.01) | <0.001 |
| Mean WSS | 14.77 (9.88-20.20) | 13.95 (10.09-19.98) | 14.70 (9.11-21.52) | 0.726 |

WSS, wall shear stress; MCA-M1, M1 segment of middle cerebral artery; P value was compared between the upstream and downstream plaque segments.

| **Absolute** **WSS measures** | Vessel curve orientation of the diseased MCA-M1 (n=176) | | | | | | |  | Tortuosity index of the diseased vessel segment (n=176) | |
| --- | --- | --- | --- | --- | --- | --- | --- | --- | --- | --- |
|  | Ventral (n=104) | Dorsal (n=72) | P value |  | Inferior (n=111) | Superior (n=65) | P value |  | Spearman R | P value |
|  |  |  |  |  |  |  |  |  |  |  |
| **Throughout the plaque** | | | | | | | | | | |
| Maximum WSS | 40.82 (23.94-59.08) | 43.09 (28.75-86.17) | 0.185 |  | 39.09 (23.76-57.19) | 46.20 (32.15-83.80) | 0.025 |  | –0.062 | 0.416 |
| Minimum WSS | 0.52 (0.25-0.79) | 0.79 (0.42-1.20) | <0.001 |  | 0.50 (0.26-0.83) | 0.79 (0.45-1.24) | <0.001 |  | –0.281 | <0.001 |
| Mean WSS | 14.53 (8.89-19.97) | 15.40 (10.93-22.26) | 0.177 |  | 14.07 (8.87-19.32) | 15.95 (11.12-23.09) | 0.032 |  | –0.055 | 0.470 |
| **Upstream plaque segment** | | | | | | | | | | |
| Maximum WSS | 37.42 (21.97-53.40) | 40.78 (26.53-75.30) | 0.112 |  | 35.38 (21.47-52.43) | 45.23 (29.13-71.07) | 0.025 |  | 0.022 | 0.776 |
| Minimum WSS | 3.13 (1.92-4.59) | 2.72 (1.76-4.82) | 0.675 |  | 3.15 (2.10-4.80) | 2.71 (1.73-4.72) | 0.427 |  | 0.018 | 0.808 |
| Mean WSS | 13.62 (9.88-19.97) | 14.06 (11.25-20.01) | 0.263 |  | 13.30 (9.92-19.53) | 15.45 (10.96-21.23) | 0.114 |  | 0.151 | 0.045 |
| **Downstream plaque segment** | | | | | | | | | | |
| Maximum WSS | 39.87 (22.96-58.81) | 39.23 (27.55-73.86) | 0.315 |  | 36.99 (22.73-53.07) | 43.24 (29.85-69.76) | 0.046 |  | –0.114 | 0.133 |
| Minimum WSS | 0.52 (0.25-0.85) | 0.83 (0.44-1.26) | <0.001 |  | 0.52 (0.26-0.87) | 0.79 (0.45-1.35) | 0.001 |  | –0.302 | <0.001 |
| Mean WSS | 13.69 (8.46-20.58) | 15.16 (10.87-21.85) | 0.146 |  | 14.01 (8.22-20.58) | 15.73 (11.16-25.22) | 0.018 |  | 0.225 | 0.003 |

**Supplemental Table 3.** Associations between the geometric features of MCA-M1 and absolute WSS measures

WSS, wall shear stress; MCA-M1, M1 segment of middle cerebral artery

**Supplemental Table 4.** Associations between morphological features of the MCA-M1 plaques and absolute WSS measures

| **Absolute** **WSS measures** | Percentage of  luminal stenosis (n=176) | |  | Plaque length  (n=176) | |  | Lesion asymmetry in the longitudinal axis  (n=176) | | |
| --- | --- | --- | --- | --- | --- | --- | --- | --- | --- |
|  | Spearman R | P value |  | Spearman R | P value |  | Upstream-dominant (n=93) | Downstream-dominant  (n=83) | P value |
| **Throughout the stenotic lesion** | | | | | | | | | |
| Maximum WSS | 0.527 | <0.001 |  | 0.081 | 0.285 |  | 48.04 (32.93-85.57) | 35.66 (21.69-50.61) | <0.001 |
| Minimum WSS | 0.295 | <0.001 |  | –0.053 | 0.481 |  | 0.64 (0.35-1.01) | 0.54 (0.26-0.86) | 0.196 |
| Mean WSS | 0.408 | <0.001 |  | 0.057 | 0.452 |  | 16.63 (11.46-23.24) | 12.91 (8.65-18.12) | <0.001 |
| **Upstream plaque segment** | | | | | | | | | |
| Maximum WSS | 0.489 | <0.001 |  | 0.050 | 0.510 |  | 46.60 (30.54-73.70) | 29.22 (19.86-47.85) | <0.001 |
| Minimum WSS | 0.093 | 0.221 |  | 0.056 | 0.460 |  | 3.15 (1.75-4.85) | 2.93 (1.86-4.45) | 0.906 |
| Mean WSS | 0.334 | 0.000 |  | 0.013 | 0.861 |  | 15.85 (11.58-21.51) | 12.45 (9.33-17.87) | 0.001 |
| **Downstream plaque segment** | | | | | | | | | |
| Maximum WSS | 0.530 | <0.001 |  | 0.110 | 0.146 |  | 46.05 (31.29-78.31) | 34.97 (21.69-48.51) | 0.001 |
| Minimum WSS | 0.291 | <0.001 |  | –0.066 | 0.382 |  | 0.69 (0.35-1.03) | 0.57 (0.26-0.95) | 0.162 |
| Mean WSS | 0.432 | <0.001 |  | –0.101 | 0.184 |  | 15.83 (11.43-23.78) | 11.94 (7.55-18.67) | 0.001 |

MCA-M1, M1 segment of middle cerebral artery; WSS, wall shear stress.
